# Brainstem sources of cardiac vagal tone and respiratory sinus arrhythmia

**DOI:** 10.1113/JP273164

**Published:** 2016-12-14

**Authors:** David G.S. Farmer, Mathias Dutschmann, Julian F.R. Paton, Anthony E. Pickering, Robin M. McAllen

**Affiliations:** ^1^ Florey Institute of Neuroscience and Mental HealthUniversity of MelbourneVictoriaAustralia; ^2^ School of PhysiologyPharmacology & NeuroscienceBiomedical SciencesUniversity of BristolBristolUK

## Abstract

**Key points:**

- Cardiac vagal tone is a strong predictor of health, although its central origins are unknown.
- Respiratory‐linked fluctuations in cardiac vagal tone give rise to respiratory sinus arryhthmia (RSA), with maximum tone in the post‐inspiratory phase of respiration.
- In the present study, we investigated whether respiratory modulation of cardiac vagal tone is intrinsically linked to post‐inspiratory respiratory control using the unanaesthetized working heart‐brainstem preparation of the rat.
- Abolition of post‐inspiration, achieved by inhibition of the pontine Kolliker‐Fuse nucleus, removed post‐inspiratory peaks in efferent cardiac vagal activity and suppressed RSA, whereas substantial cardiac vagal tone persisted. After transection of the caudal pons, part of the remaining tone was removed by inhibition of nucleus of the solitary tract.
- We conclude that cardiac vagal tone depends upon at least 3 sites of the pontomedullary brainstem and that a significant proportion arises independently of RSA.

**Abstract:**

Cardiac vagal tone is a strong predictor of health, although its central origins are unknown. The rat working heart‐brainstem preparation shows strong cardiac vagal tone and pronounced respiratory sinus arrhythmia. In this preparation, recordings from the cut left cardiac vagal branch showed efferent activity that peaked in post‐inspiration, ∼0.5 s before the cyclic minimum in heart rate (HR). We hypothesized that respiratory modulation of cardiac vagal tone and HR is intrinsically linked to the generation of post‐inspiration. Neurons in the pontine Kölliker‐Fuse nucleus (KF) were inhibited with bilateral microinjections of isoguvacine (50–70 nl, 10 mm) to remove the post‐inspiratory phase of respiration. This also abolished the post‐inspiratory peak of cardiac vagal discharge (and cyclical HR modulation), although a substantial level of activity remained. In separate preparations with intact cardiac vagal branches but sympathetically denervated by thoracic spinal pithing, cardiac chronotropic vagal tone was quantified by HR compared to its final level after systemic atropine (0.5 μm). Bilateral KF inhibition removed 88% of the cyclical fluctuation in HR but, on average, only 52% of the chronotropic vagal tone. Substantial chronotropic vagal tone also remained after transection of the brainstem through the caudal pons. Subsequent bilateral isoguvacine injections into the nucleus of the solitary tract further reduced vagal tone: remaining sources were untraced. We conclude that cardiac vagal tone depends on neurons in at least three sites of the pontomedullary brainstem, and much of it arises independently of respiratory sinus arrhythmia.

## Key points

- Cardiac vagal tone is a strong predictor of health, although its central origins are unknown.
- Respiratory‐linked fluctuations in cardiac vagal tone give rise to respiratory sinus arryhthmia (RSA), with maximum tone in the post‐inspiratory phase of respiration.
- In the present study, we investigated whether respiratory modulation of cardiac vagal tone is intrinsically linked to post‐inspiratory respiratory control using the unanaesthetized working heart‐brainstem preparation of the rat.
- Abolition of post‐inspiration, achieved by inhibition of the pontine Kolliker‐Fuse nucleus, removed post‐inspiratory peaks in efferent cardiac vagal activity and suppressed RSA, whereas substantial cardiac vagal tone persisted. After transection of the caudal pons, part of the remaining tone was removed by inhibition of nucleus of the solitary tract.
- We conclude that cardiac vagal tone depends upon at least 3 sites of the pontomedullary brainstem and that a significant proportion arises independently of RSA.

**Abbreviations**
CVBA: cardiac vagal branch activity
DMNV: dorsal motor nucleus of vagus
HR: heart rate
KF: Kölliker‐Fuse nucleus
NA: nucleus ambiguus
NTS: nucleus tractus solitarii
PNA: phrenic nerve activity
RSA: respiratory sinus arrhythmia
WHBP: working heart‐brainstem preparation

## Introduction

From at least the middle of the 19th century onward, it has been known that the vagal supply to the heart tonically holds down heart rate (HR) (Weber, 1846; Bezold, 1858). Additionally, vagal activity provides control of atrioventricular conduction (Massari *et al*. 1995) and ventricular excitability and contractility (Machhada *et al*. 2015, 2016). The central origins of cardiac vagal tone remain essentially unknown.

The principal preganglionic vagal motoneurons that regulate HR have myelinated axons (B fibres) and are located mainly in the external formation of the nucleus ambiguus (NA) (McAllen & Spyer, 1976; Nosaka *et al*. 1979, 1982; Hopkins *et al*. 1996). In slice preparations of the medulla *in vitro*, vagal motorneurons in the NA identified by retrograde transport of dye from the epicardium show little or no ongoing spike activity (Mendelowitz, 1996; Dergacheva *et al*. 2010). Tonic activity of vagal motoneurons *in vivo* therefore presumably arises from ongoing synaptic input, driven directly or indirectly from areas of the brain that are disconnected in that preparation. Numerous projections from the medulla, pons and hypothalamus to cardiac vagal motorneurons (or regions of the NA known to contain them) have been identified (Herbert *et al*. 1990; Standish *et al*. 1995; Neff *et al*. 1998; Piñol *et al*. 2012; Poon & Song, 2014), although it remains unclear which (if any) contribute to ongoing cardiac vagal tone.

Cells in the other vagal preganglionic nucleus (DMNV) have unmyelinated axons (Cheng *et al*. 2004). Although electrical stimulation of unmyelinated vagal efferents in cats and rats can cause modest slowing of the heart (Jones *et al*. 1995), electrical stimulation of DMNV in cats was not found to slow the heart (Geis & Wurster, 1980) The main cardiac function of DMNV neurons is to exert inhibitory control of the excitability and inotropic state of the ventricles (Geis & Wurster, 1980). Accordingly, selective pharmacogenetic inhibition of DMNV neurons *in vivo* was found to alter ventricular function but not HR (Machhada *et al*. 2015, 2016), indicating that the DMNV does not provide tone to the cardiac pacemaker.

Variations in vagal chronotropic drive give rise to the respiratory sinus arrhythmia (RSA), raising HR in inspiration and decreasing it during expiration (Japundzic *et al*. 1990). Sympathetic effects on HR are too sluggish to contribute significantly to the RSA (Warner & Cox, 1962). Hypotheses concerning the physiological significance of the RSA include optimization of ventilation–perfusion matching in the lung, minimization of cardiac energy expenditure and optimal maintenance of blood CO_2_ partial pressure (Yasuma & Hayano, 2004; Sin *et al*. 2010; Ben‐Tal *et al*. 2012). Clinically, ‘high frequency’ or beat‐to‐beat HR variability, which are essentially measures of RSA, are regarded as a direct index of ongoing cardiac vagal tone (Chess *et al*. 1975; Akselrod *et al*. 1981; Pomeranz *et al*. 1985; Pagani *et al*. 1986). Reduced RSA is an independent predictor of mortality in chronic heart failure and after myocardial infarction (La Rovere *et al*. 1998, 2003). It is clear that both central and reflex mechanisms contribute to this waxing and waning of vagal tone over the respiratory cycle (Anrep *et al*. 1936 *a*, *b*). The relative contributions of each are still a matter of debate (Taha *et al*. 1995; Eckberg, 2009; Karemaker, 2009), although it is agreed that there is an important central component linked to central respiratory drive (Anrep *et al*. 1936 *b*; Eckberg *et al*. 1980; Gilbey *et al*. 1984; Taha *et al*. 1995; Simms *et al*. 2007).

It is accepted that the ‘kernel’ of the respiratory rhythm generator is within the ventral respiratory column of the medulla (Feldman & Del Negro, 2006; Ramirez *et al*. 2012; Smith *et al*. 2013). The generation of a three‐phased, eupnoeic respiratory motor ‘pattern’, however, depends upon the integrity of connections between the medulla and pons (Markwald, 1887; Lumsden, 1923; Rybak *et al*. 2004, 2007; Dutschmann & Dick, 2012; Poon & Song, 2014). Transection and lesion studies using the *in situ* perfused working heart and brainstem preparation of the rat (WHBP) have demonstrated that pontine nuclei are essential to the expression of the post‐inspiratory phase (Dutschmann & Herbert, 2006; Smith *et al*. 2007). In the absence of these nuclei, synaptic mechanisms leading to the termination of the inspiratory phase (the ‘inspiratory off‐switch’) are delayed: spontaneous inspiratory phrenic nerve discharge takes the form of an apneustic ‘square‐wave’ (Smith *et al*. 2007) and sequential activation of the motor outputs that drive secondary respiratory muscles (e.g. the laryngeal adductor muscles) is abolished. Focal inhibition of the pontine Kölliker‐Fuse nucleus (KF) also abolishes post‐inspiration, suggesting that this is a crucial locus of pontine post‐inspiratory control (Rybak *et al*. 2004; Dutschmann & Herbert, 2006; Smith *et al*. 2007; Dutschmann & Dick, 2012; Bautista & Dutschmann, 2014).

Studies on vagal tone have been significantly hampered by the use of general anaesthesia and invasive investigations. To a greater or lesser degree, anaesthetics suppress vagal tone (Inoue & Arndt, 1982), making its origins difficult to study. The WHBP, which is decerebrate but unanaesthetized, offers a simplified preparation that shows abundant cardiac vagal tone with a physiological pattern of RSA (Paton, 1996; Simms *et al*. 2007; Pickering *et al*. 2008). Under the conditions of the WHBP, cardiac vagal branch activity (CVBA) peaks, and HR is lowest, in the post‐inspiratory period (Simms *et al*. 2007; Pickering *et al*. 2008). Furthermore, transection of the pons, which separates the respiratory kernel from the KF, abolishes RSA (Baekey *et al*. 2008). Based on these data, we hypothesized that respiratory modulation of cardiac vagal tone and HR is intrinsically linked to the generation of post‐inspiration. We predicted that focal inhibition of the KF would abolish respiratory‐linked fluctuations in cardiac vagal activity, as well as reduce, or perhaps abolish, cardiac vagal tone. We have investigated the ongoing contributions of selected pontine and medullary nuclei, revealing their common and distinct influences on RSA and on chronotropic vagal drive.

## Methods

All experimental procedures were performed in accordance with the Australian code of practice for the care and use of animals for scientific purposes and conform with the principles of international regulations. This study was approved by, and carried out in accordance with guidelines put in place by the ethics committee of the Florey Institute of Neuroscience and Mental Health, Melbourne, Australia.

### WHBP

Experiments were performed using the arterially perfused *in situ* brainstem preparation (Paton, 1996) and used juvenile Sprague–Dawley rats of either sex, aged 17–29 days. All basic procedures performed were conducted in accordance with the protocols described previously (Dutschmann & Herbert, 2006; Farmer *et al*. 2014). Briefly, juvenile rats were anaesthetized with isoflurane, bisected below the diaphragm and immersed in ice‐cold Ringer solution (in mm): 125 NaCl, 24 NaHCO_3_, 2.5  CaCl_2_, 1.25 MgSO_4_, 4 KCl, 1.25 KH_2_PO_4_ and 10 d‐glucose and 1.25% ficoll; Sigma, St Louis, MO, USA). Animals were decerebrated at the level of the superior colliculus and cerebellectomized to expose the brainstem. The lungs were excised, leaving a small amount of parenchymal tissue around the cut left bronchus. The thymus was resected to gain access to the left cardiac vagal branch. Taking care to preserve the left phrenic nerve, the diaphragm was resected, except for a small part around the oesophagus, which was tied to a length of silk thread. The left phrenic nerve was isolated, ready for recording. Preparations were transferred to a recording chamber and perfused via the descending aorta with Ringer solution bubbled with 95% O_2_ and 5% CO_2_ (carbogen) at 31ºC. Phrenic nerve activity was recorded with a suction electrode and this signal was used when determining the appropriate perfusion flow and pressure values to obtain a eupnoeic pattern of central respiratory drive. The eupnoeic pattern consisted of spontaneous rhythmic discharges lasting ≤1 s, showing a ramped onset and a rapid termination (Paton, 1996; Pickering & Paton, 2006). In different preparations, this required final flow rates of 18–22 ml min^−1^. In some experiments, a bolus of sodium cyanide (0.1 ml, 0.1%) was added to the perfusate to stimulate arterial chemoreceptors. Similarly, arterial baroreceptors were activated by transiently (2–3 s), setting the perfusion pump to its maximum setting (Paton & Butcher, 1998).

### Nerve recording

Activity was recorded from the cut proximal ends of isolated nerves using suction electrodes. In all experiments, phrenic nerve activity (PNA) was recorded monopolarly with respect to the bath ground electrode. The ECG was recorded from the ear bars used to immobilize the animal or from an electrode placed directly on the surface of the myocardium.

### Quantification of cardiac vagal tone

#### Nerve recordings

To record from the cardiac vagal branch, the animal was positioned semi‐prone and the oesophagus gently retracted caudally by its attached thread. The left thoracic vagus nerve could be observed parallel to the oesophagus by manipulation of the left bronchial root. The thoracic vagus was carefully dissected away from the surrounding tissue to expose its branches. The left cardiac vagus branch was usually found just caudal to the left recurrent laryngeal nerve, projecting between the left precava and pulmonary artery to innervate the heart (Burkholder *et al*. 1992; Simms *et al*. 2007). When necessary, these vessels were removed to improve access. The cardiac vagal branch was then separated from the surrounding tissue and cut as distally as possible. Cardiac vagal branch activity (CVBA) was recorded differentially between a fine silver wire placed at the origin of the cardiac branch and a suction electrode distally. Suction electrode tips were broken to size and, once in the electrode, the cardiac vagal branch was lifted from the surrounding circulating fluid into the air. Nerve signals were amplified (differential amplifier DP‐311; Warner Instruments, Hamden, USA; Gain: 1000–10 000×), band‐pass filtered (100 Hz to 5 kHz), digitized at 2 or 5 kHz (PowerLab/16SP; ADInstruments, Sydney, Australia) and recorded using LabChart, version 7/8 (ADInstruments, Sydney, Australia).

The viability of the cardiac vagal branch recording was tested with an excitatory baroreceptor challenge by briefly (2–3 s) increasing the perfusion pump setting to its maximum, taking care that rising fluid levels caused no artefact. Preparations that failed this test were either repositioned until the test was successful or discarded. At the end of each experiment, the perfusion pump or the carbogen gas was switched off and recordings continued until nerve activity ceased. The residual noise level was used to determine the threshold for subsequent analysis of discriminated nerve activity.

#### HR

In some experiments, the cardiac vagal branches were left intact and fluctuations in cardiac vagal tone were assessed indirectly as changes in HR. In these experiments, the thoracic spinal cord was destroyed by pithing with a 16‐G needle repeatedly inserted into the spinal cavity via the distal end of the severed spinal cord. In each case, the required length of needle was determined using the spinal processes of the proximal thoracic vertebrae as a measure prior to insertion. Thus, the influence of the sympathetic nervous system was effectively removed at the same time as ensuring that the phrenic motoneurons, located in the caudal cervical spinal cord, were not damaged (as indicated by preserved phrenic nerve activity).

### Microinjections, transections and denervations

Local microinjection of drugs (50–70 nl) was performed using three‐barrel borosilicate glass micropipettes, broken to a tip diameter of ∼ 20 μm. The individual barrels were filled with l‐glutamate (10 mm in saline), the GABA_A_ receptor agonist isoguvacine (10 mm in saline) or Chicago sky blue (2% in glucose‐free Ringer solution). The volume of each microinjection was monitored microscopically by movement of the liquid meniscus in relation to a calibrated scale attached to the pipette. Landmarks on the dorsal surface of the brainstem were used to identify the rostrocaudal and mediolateral co‐ordinates of the KF. As described previously (Dutschmann & Herbert, 2006), unilateral stimulation of the KF with l‐glutamate produces an extension of the post‐inspiratory phase, and this effect was used to select sites for subsequent inhibition with isoguvacine. If bilateral isoguvacine injections failed to prolong inspiration by > 200%, they were regarded to have missed the critical region (Dutschmann & Herbert, 2006) and the data were excluded. In several control experiments, isoguvacine was injected at a pontine site 1 mm medial to the KF after its identification by l‐glutamate injection. In all cases, isoguvacine injection sites were marked by pressure‐injecting ∼100 nl Chicago sky blue.

Caudal pontine transections were performed using the caudal extent of the cerebellar paraflocculi as a reference point. A scalpel blade was inserted downwards with the cutting edge at a rostral angle of ∼10° such that the brain stem was transected at a level near the rostral pole of the facial nucleus.

After pontine transection, nucleus tractus solitarii (NTS) neurons were inhibited by 2 × 100 nl bilateral microinjections of isoguvacine at two rostrocaudal levels: the first at the level of the calamus scriptorius, 300 μm lateral to midline and 500 μm ventral to the brain surface; and the second, 500 μm rostral to calamus scriptorius, 500 μm lateral and 500 μm ventral.

In six experiments, the contributions of peripheral chemoreceptor and/or baroreceptor afferents to ongoing cardiac vagal tone were assessed by cutting the carotid sinus and aortic depressor nerves (Pickering *et al*. 2008). The abolition of previously intact responses to raised perfusion pressure and to sodium cyanide was taken as confirmation of successful denervation. It was often necessary to sever the superior laryngeal nerves to complete the aortic baroreceptor denervation.

### Histological assessment of microinjection sites and transections

Brainstems were immersion‐fixed in 4% paraformaldehyde for several days, transferred to 30% sucrose in PBS for at least 24 h and then serially sectioned at 50 μm using a freezing microtome and counterstained with neutral red. The locations of microinjections, as indicated by Chicago sky blue, were documented on schematic drawings of coronal sections containing the KF. The level and completeness of transections were confirmed by examination of sagittal sections.

### Statistical analysis

Data were exported from Lab Chart as text files and imported into Spike2 (Cambridge Electronic Design, Cambridge, UK). Instantaneous HR (beats min^–1^) was calculated using events triggered by the P wave of the atrial ECG because this provides the least confounded measure of chronotropic vagal tone. To quantify CVBA, action potentials rising above the noise threshold (established at the end of the experiment as described above) were counted. Mean values for HR and CVBA frequency were generated over representative sections of the experimental trace encompassing at least 10 respiratory cycles. Over the same period, event‐triggered means (triggered by the rapid fall in phrenic activity which occurs at the end of inspiration) of HR and CVBA were generated and displayed as multiples of the mean frequency over the baseline averaging period. The magnitude of the RSA was defined as the difference between the maximum and minimum HR as calculated from the event‐triggered mean. Similarly, the magnitude of respiratory‐linked fluctuations in CVBA was calculated as the difference between the maximum and minimum frequencies of firing.

In experiments where CVBA was recorded, the effects of baroreceptor activation and unilateral microinjection of glutamate into KF on CVBA and HR were quantified. Peak CVBA (time constant = 0.25 s) was averaged over five respiratory cycles prior to baroreceptor activation or injection of glutamate, and then compared with peak CVBA immediately following the stimulus. The magnitude of bradycardia was assessed by comparison of the mean HR over the same five respiratory cycles with the minimum value observed after the stimulus. In the case of KF glutamate microinjections, the total duration of the respiratory cycle (i.e. the time between onset of a spontaneous phrenic nerve burst and the onset of the next) immediately following microinjection was compared with the mean duration of the five preceding respiratory cycles.

Chemoreceptor activation of the cardiac vagal branch was assessed by comparing mean peak CVB firing frequency (over five respiratory cycles prior to the administration of 0.1 ml of 0.1% sodium cyanide) with peak CVB firing frequency over the four or five respiratory cycles that followed.

Statistical analyses were carried out using the R statistical programming environment (Rstudio, version 0.99.902; RStudio Inc., Boston, MA, USA). The normality of each data set was established using the Shapiro–Wilk test for normality. Where more than two treatments were compared, data were analysed using a repeated measures ANOVA before *post hoc* analyses. The statistical significance of changes in mean HR, magnitude of RSA, respiratory cycle duration, mean CVBA and respiratory fluctuations in CVBA were assessed by a paired *t* test. Where appropriate, *P* values were adjusted for multiple comparisons using the Bonferroni method.

## Results

**Figure 1:**
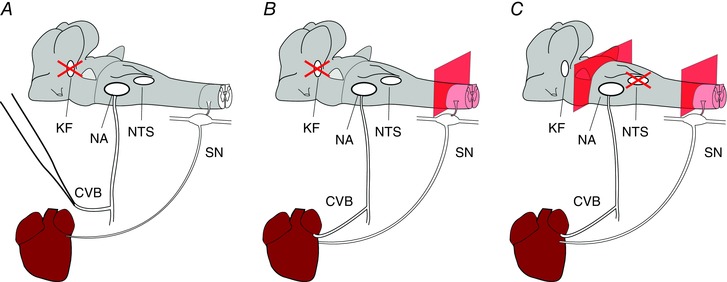
Variations on the WHBP used to investigate the origins of cardiac vagal tone Three variations of WHBP were used in the present study. Red crosses indicate sites of microinjections. In the first (*A*), a cardiac branch of the left thoracic vagus was isolated and severed. This allowed direct, differential recordings of electrical activity of the neurons that project to the heart within this branch. Because making these recordings required that we unilaterally dennervate the heart, these experiments were repeated in preparations with cardiac vagal branches left intact (*B*). In this case, the thoracic spinal cord was destroyed, removing the confounding chronotopic influence of sympathetic cardiac nerves. Finally (*C*), the relative contribution of pontine and medullary sources of cardiac vagal tone was assessed by transection of the brainstem at the level of the caudal pons. In each case, the atrial ECG was recorded from the surface of the atrium. The HR was calculated using the interval between adjacent P waves. CVB, cardiac vagal branch; KF, Kölliker‐Fuse; NA, nucleus ambiguus; NTS, nucleus tractus solitarii; SN, cardiac sympathetic nerve. [Colour figure can be viewed at wileyonlinelibrary.com]

Experiments were performed on three variations of the rat WHBP (Fig. 1).

### Cardiac vagal branch recording

**Figure 2:**
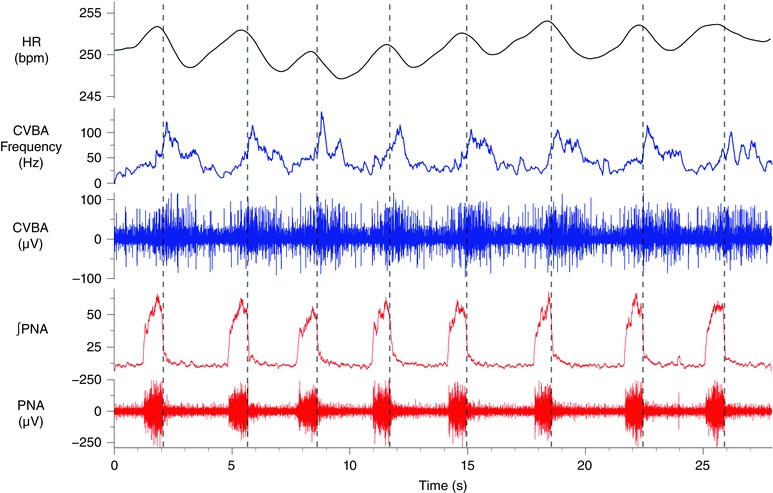
Spontaneous phrenic discharge, respiratory‐phasic CVBA and RSA in the rat WHBP A representative trace from one of twelve animals in which cardiac vagal branch recordings were made. Vertical broken lines mark the rapid decline in PNA (both raw nerve activity and the integrated waveform are depicted) that denotes the onset of post‐inspiration. CVBA (both raw nerve activity and mean frequency of firing are depicted) showed marked respiratory modulation: increasing during the inspiratory period and peaking in the post‐inspiratory period before falling to a minimal level in late expiration. These preparations also displayed a clear RSA. ∫PNA, integrated PNA. [Colour figure can be viewed at wileyonlinelibrary.com]

Cardiac vagal branch recordings were made in 12 preparations (Fig. 2). Even though the left cardiac vagal branch had been cut, these preparations displayed a clear RSA (mean difference in maximum – minimum HR over one respiratory cycle of 8.9 ± 2.1 beats min^–1^). CVBA showed marked respiratory modulation and increased during the inspiratory period with minimal discharge in late expiration. Respiratory cycle‐triggered means of CVBA and HR confirmed in 11 of 12 preparations that CVBA increased during inspiration and peaked immediately after the termination of phrenic discharge, in the post‐inspiratory phase. Peak cardiac vagal branch activity preceded minimum HR by 527 ± 43 ms.

**Figure 3:**
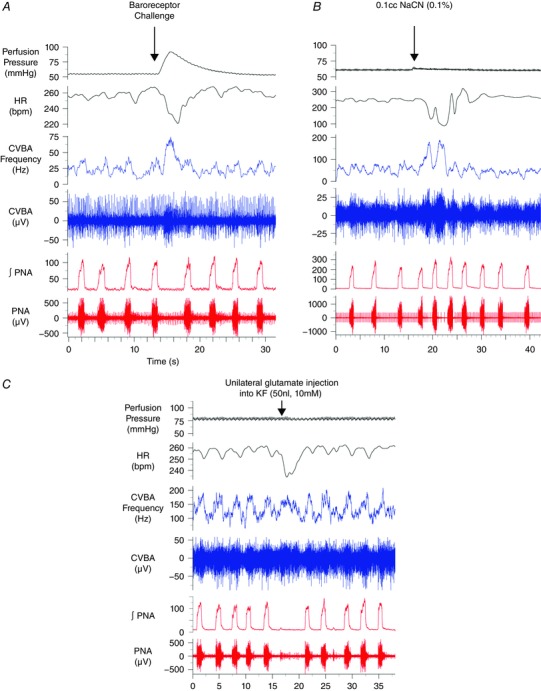
Reflex activation and KF stimulation: effects on CVBA Representative traces from three separate experiments. Baroreceptor challenges, produced by a transient increase in the perfusion pressure (*A*), were associated with marked activation of the cardiac vagal branch and bradycardia. A bolus of sodium cyanide added to the perfusing solution (0.1 ml, 0.1%) evoked an increase in the magnitude and frequency of phrenic discharge and enhanced the firing rate of the cardiac vagal branch during post‐inspiration (*B*). These post‐inspiratory discharges were associated with periods of bradycardia. Unilateral microinjection of 10 mm glutamate into the KF prolonged the expiratory period (*C*). CVBA was maintained during the extended expiratory period, producing a bradycardia. ∫PNA, integrated phrenic nerve activity. [Colour figure can be viewed at wileyonlinelibrary.com]

A baroreceptor challenge produced an increase in the peak activity of the cardiac vagal branch (control: 87.1 ± 7.4 Hz; baroreceptor challenge: 135.7 ± 12.9 Hz; *P* < 0.0001) with accompanying bradycardia (control: 259.6 ± 10.2 beats min^–1^; baroreceptor challenge: 194.1 ± 16.3; *P* = 0.0004). A representative experimental trace is depicted in Fig. 3 *A*. Administration of sodium cyanide (0.1 ml, 0.1%) to the perfusate produced an increase in phrenic discharge frequency (control: 13.4 ± 1.1 breaths min^–1^; chemoreceptor challenge: 21.3 ± 1.4 breaths min^–1^; *P* < 0.0001), with enhanced post‐inspiratory cardiac vagal branch discharge (control: 83.3 ± 7.4 Hz; chemoreceptor challenge; 125.4 ± 11.1 Hz; *P* = 0.0001) and corresponding bradycardia (Fig. 3 *B*). These features were used to confirm the identity of the recorded nerve as being the cardiac vagus.

As described previously (Dutschmann and Herbert 2006), unilateral microinjection of l‐glutamate into the KF (*n* = 10; ipsilateral to the remaining, intact cardiac vagal branch) produced an increase in the respiratory period (control: 4.4 ± 0.4 s; glutamate: 13.7 ± 2.5 s; *P* = 0.002) (Fig. 3 *C*). Cardiac vagal branch activity was maintained throughout the extended post‐inspiratory period and fired at rates that were slightly higher than those observed during post‐inspiration prior to microinjection (control: 100.3 ± 10.6 Hz; glutamate: 114.3 ± 10.3 Hz; *P* = 0.015). This was also associated with a bradycardia (control: 270.3 ± 11.7 beats min^–1^; KF glutamate: 243.0 ± 17.7 beats min^–1^; *P* = 0.040).

**Figure 4:**
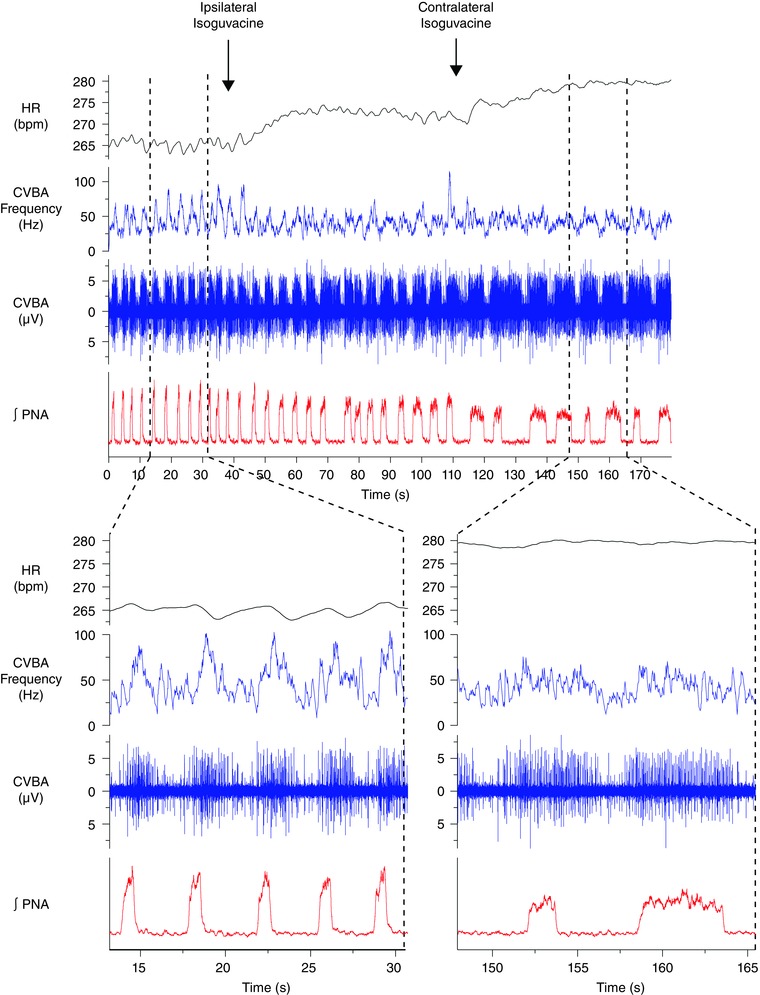
Inhibition of the KF suppresses the post‐inspiratory peak in CVBA and RSA A representative trace from a single experiment depicting sequential inhibition of the ipsilateral and contralateral KF with isoguvacine (top panel). Times of injection are indicated by labelled arrows. Sections denoted by broken lines depict time‐expanded views of the trace before and after bilateral inhibition of the KF (bottom). Bilateral inhibition of the KF produced an apneustic pattern of discharge in the phrenic nerve. The post‐inspiratory peak in CVBA was abolished along with the RSA. A mild tachycardia was observed. CVBA assumed a tonic pattern of discharge, occasionally interrupted by periods on inhibition in late expiration that were not associated with an obvious change in HR. ∫PNA, integrated PNA. [Colour figure can be viewed at wileyonlinelibrary.com]

**Figure 5:**
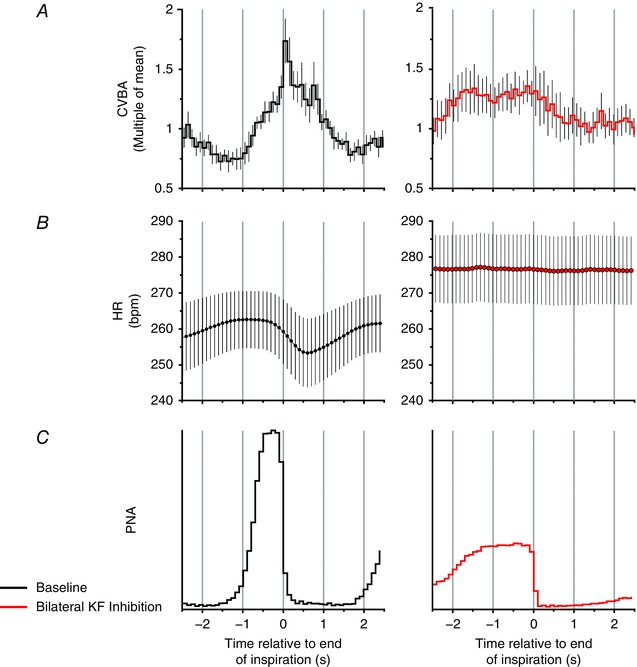
Post‐inspiratory cycle‐triggered means of CVBA and HR before and after bilateral inhibition of the KF Group data: cycle‐triggered means of CVBA (*A*), HR (*B*) and PNA (*C*). Means were triggered by the rapid decline in PNA denoting the onset of post‐inspiration. Mean CVBA at baseline and after inhibition of the KF is displayed as multiples of the mean frequency over the baseline averaging period: this comprised at least 10 respiratory cycles. At baseline (left), the post‐inspiratory peak in CVBA and the nadir in RSA were prominent. After bilateral inhibition of the KF, the post‐inspiratory peak in CVBA was suppressed, mean HR increased and RSA was no longer apparent. Data are the mean ± SEM (minimum of 10 sweeps/experiment; *n* = 8). [Colour figure can be viewed at wileyonlinelibrary.com]

**Figure 6:**
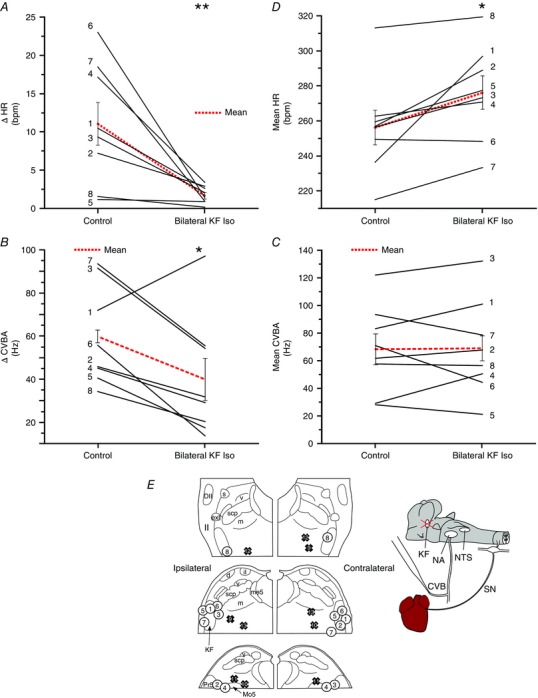
Inhibition of the KF reduces the magnitude of respiratory fluctuations in cardiac vagal activity and RSA The magnitude of the RSA (ΔHR) (*A*) and of cyclical fluctuations in CVBA (ΔCVBA) (*B*) was reduced by bilateral injection of isoguvacine into the KF. Mean HR (*C*) showed an increase, whereas mean CVBA (*D*) was unchanged. Isoguvacine injection sites for each experiment are depicted in (*E*): numbered circles depict KF injections corresponding to numbered experiments in (*A*) to (*D*); numbered crosses depict control injections made 1 mm medial to KF (data not shown; see Results). ΔHR and ΔCVNA were calculated as the difference between the maximum and the minimum values over the course the single respiratory cycle. All parameters were averaged over ≥ 10 cycles/experiment. Data are values from individual experiments (black lines) and are the mean ± SEM (red dashed lines). ^*^ *P* < 0.05, ^**^ *P* < 0.01, paired *t* test (*n* = 8). il, internal lateral parabrachial nucleus; me5, mesencephalic trigeminal tract; Pr5, principal sensory trigeminal nucleus; scp, superior cerebellar peduncle m, medial parabrachial nucleus; s, superior lateral parabrachial nucleus; v, ventral lateral parabrachial nucleus. [Colour figure can be viewed at wileyonlinelibrary.com]

Bilateral inhibition of the KF with isoguvacine altered the pattern of phrenic nerve discharge to one of apneusis (*n* = 8) (Figs 4 and 5) as described previously (Dutschmann & Herbert, 2006). The post‐inspiratory peak in CVBA activity disappeared, although brief dips in activity occurring in expiration sometimes persisted. These dips, when present, were not sufficient to induce any measurable tachycardia (Figs 5 and 6). Group mean data are shown in Figs 5 and 6.

RSA in these preparations was reduced from a mean of 11.0 ± 2.8 beats min^–1^ to 1.7 ± 0.4 beats min^–1^ (*P* = 0.0012) (Fig. 6 *A*) after bilateral KF isoguvacine injections (Figs 4, 5, 6) and the magnitude of respiratory‐linked fluctuations in CVBA was reduced (baseline: 59.8 ± 8.1 Hz; KF isoguvacine: 39.9 ± 9.9 Hz; *P *= 0.035) (Fig. 6 *B*). Mean HR increased from 256.2 ± 9.8 beats min^–1^ to 276.0 ± 9.6 beats min^–1^ (*P* = 0.022) (Fig. 6 *C*). Mean CVBA was unchanged (baseline: 64.3 ± 12.3 Hz; isoguvacine: 66.6 ± 13.5 Hz; *P* = 0.642) (Fig. 6 *D*). Histological examination of the injections sites confirmed that all injections were in the immediate vicinity of the KF (Fig. 6 *E*, numbered circles).

Bilateral control injections of isoguvacine were made 1 mm medial to the KF in five other rats (Fig. 6 *E*, numbered crosses). These did not significantly alter mean HR (control: 294.2 ± 22.6 beats min^–1^; isoguvacine: 288.0 ± 23.1 beats min^–1^; *P* = 0.159), RSA (control: 4.1 ± 1.1 beats min^–1^; isoguvacine: 5.9 ± 2.2 beats min^–1^; *P* = 0.466), mean CVBA (control: 82.2 ± 14.8 Hz; isoguvacine: 88.4 ± 17.3 Hz; *P* = 0.417) or the magnitude of respiratory‐linked fluctuations in CVBA (control: 32.6 ± 2.6 Hz; isoguvacine: 30.18 ± 3.2 Hz; *P* = 0.198).

### Cardiac vagal tone in WHBP with intact vagi (and without sympathetic drive)

**Figure 7:**
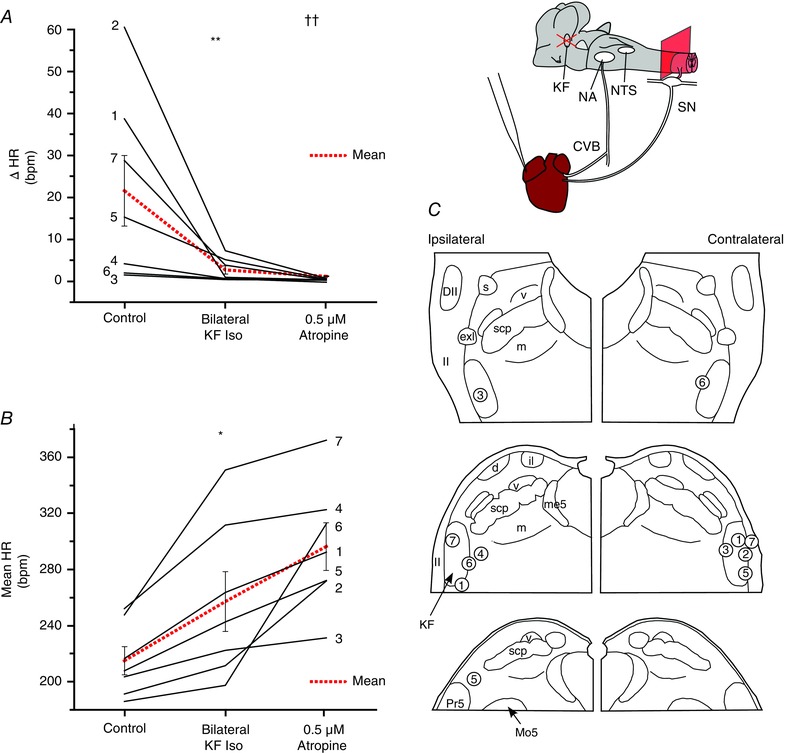
Cardiac vagal tone persists after inhibition of the KF in sympathetically‐disabled preparations Isoguvacine injections were repeated in seven animals with intact cardiac vagal branches and whose cardiac sympathetic innervation was disabled by destruction of the thoracic spinal cord. Cardiac vagal tone was quantified by comparing the HR before and after isoguvacine injection with its final level after systemic atropine. Inhibition of the KF greatly reduced the magnitude of the RSA (ΔHR) (*A*). The addition of atropine to the perfusing solution removed any remaining HR fluctuations. On average, inhibition of the KF removed half of chronotropic vagal tone (*B*). Isoguvacine injection sites for each experiment (numbered circles) (*C*). Data are values from individual experiments (black lines) and are the mean ± SEM (red dashed lines). ^**^ *P* < 0.01 compared to control; ^***^ *P* < 0.001 compared to control, ^††^ *P* < 0.01 compared to KF isoguvacine pairwise *t* test (*n* = 7). il, internal lateral parabrachial nucleus; me5, mesencephalic trigeminal tract; Pr5, principal sensory trigeminal nucleus; scp, superior cerebellar peduncle m, medial parabrachial nucleus; s, superior lateral parabrachial nucleus; v, ventral lateral parabrachial nucleus. [Colour figure can be viewed at wileyonlinelibrary.com]

Because CVBA may include action potentials of neurons that are not involved in chonotropic control and, because recording CVBA requires unilateral vagal denervation of the heart, parallel experiments were performed by measuring HR in WHBP with intact cardiac vagal connections. To avoid any confounding action of sympathetic nerves, the sympathetic outflow was disabled by destruction of the thoracic spinal cord (Fig. 7). Evidence for an effect of the following treatments upon both the magnitude of RSA and HR was found (*P* = 0.0001 and 0.0069, respectively; one‐way repeated measures ANOVA). In seven of these preparations, bilateral inhibition of KF neurons with isoguvacine reduced RSA from 20.7 ± 8.1 beats min^–1^ to 2.5 ± 1.0 beats min^–1^ (*P* = 0.001) and increased HR (baseline: 214.6 ± 9.6 beats min^–1^; after isoguvacine: 256.1 ± 20.8 beats min^–1^; *P* = 0.025). Subsequent systemic administration of atropine (0.5 μM) further reduced RSA to 0.3 ± 0.1 beats min^–1^ (*P* = 0.0044 compared to KF isoguvacine). This resulted in a further increase in HR to 294.7 ± 16.7 beats min^–1^ (*P* = 0.065 compared to KF isoguvacine). Thus, inhibition of KF bilaterally removed ∼52% of the vagal chronotropic tone. Examination of histological samples confirmed that all injections were in the immediate vicinity of the KF (Fig. 7 *C*) with two exceptions: in animal 2, the ipsilateral injection was found to be in the intertrigeminal region, caudal and ventral to KF; the contralateral injection site for animal 4 could not be recovered. These results were included because they evidently spread sufficiently to the KF to fulfil the physiological criteria for a successful injection (see Methods).

**Figure 8:**
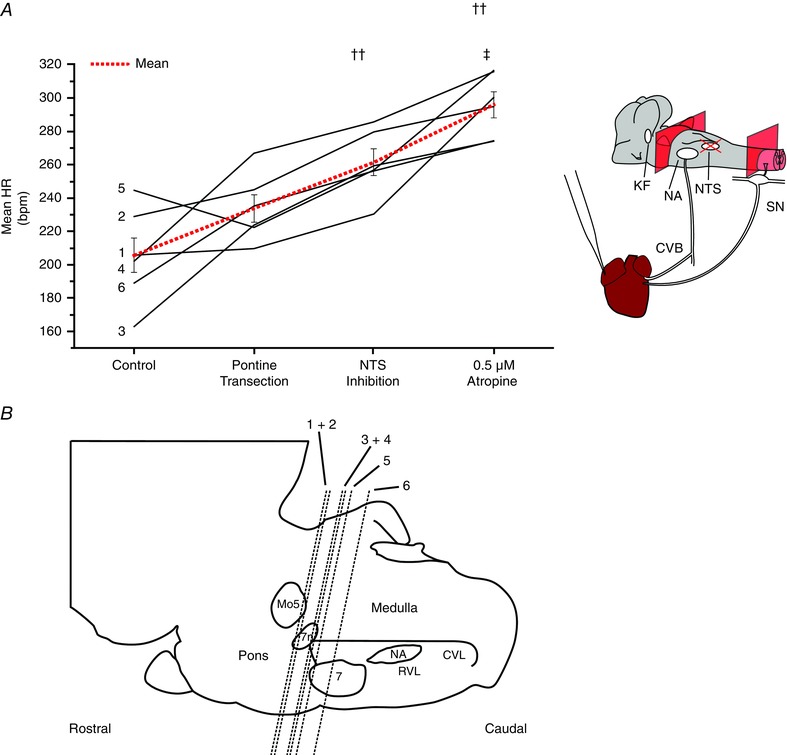
Pontine and medullary sources of cardiac vagal tone Cardiac vagal tone was again quantified by comparing the HR before the removal of suspected sources of cardiac vagal tone with its final level after systemic atropine. The magnitude and direction of changes in HR produced by pontine transection varied greatly between experiments, indicating the presence of both excitatory and inhibitory influences on cardiac vagal tone (*A*). Substantial cardiac vagal tone survived this procedure. Subsequent inhibition of the NTS produced an increase in HR indicating that this is a source of cardiac vagal drive. The level of transection from each experiment can be seen in (*B*). Data are values from individual experiments (black lines) and are the mean ± SEM (red dashed lines)., ††*P* < 0.01 compared to pontine transection; ‡*P* < 0.05 compared to NTS isoguvacine, paired *t* test (*n* = 6). 7, facial nucleus; 7n, facial nerve; CVL, caudal ventrolateral medulla; Mo5, motor trigeminal nucleus; RVL, rostral ventrolateral medulla. [Colour figure can be viewed at wileyonlinelibrary.com]

To track down remaining sources of cardiac vagal tone, the caudal pons was transected in six other sympathetically‐disabled preparations before inhibition of the NTS. Evidence for an effect of the following treatments upon HR was found (*P* < 0.0001; repeated measures ANOVA). Pontine transection produced HR changes of variable magnitude and direction (baseline: 205.6 ± 10.2 beats min^–1^; pontine transection: 233.9 ± 8.2 beats min^–1^, *P* = 0.307) (Fig. 8 *A*), although substantial vagal tone remained (final HR after atropine: 296.1 ± 7.7 beats min^–1^; *P* = 0.0041 compared to pontine transection) (Fig. 8 *A*). Approximately half of that tone was removed by inhibition of the NTS (HR after pontine transection and inhibition of the NTS: 261.5 ± 8.0 beats min^–1^; *P* = 0.0012 compared to HR after atropine) (Fig. 8 *A*).

To test whether any of the NTS‐dependent component of vagal tone could be attributed to ongoing afferent inputs from arterial chemoreceptors or baroreceptors, we performed denervation experiments in six further sympathetically‐disabled preparations. Bilateral section of the carotid sinus and aortic depressor nerves caused no increase in HR (baseline: 206.1 ± 20.6 beats min^–1^; denervated: 198.2 ± 23.0 beats min^–1^; *P* = 0.4573). In all cases, effective denervation was confirmed by abolition of baroreceptor and chemoreceptor reflexes (not shown).

Finally, and in these same preparations, the possibility that some vagal tone might arise from within the cardiac ganglion itself was addressed. The cervical vagi were cut bilaterally and, once the new baseline HR had stabilized, atropine was added to the perfusate. This did not increase HR (vagotomized: 264.0 ± 11.1 beats min^–1^; atropine: 259.1 ± 10.7; *P* = 0.162).

## Discussion

The WHBP shows a consistent pattern of RSA, with minimum HR occurring in post‐inspiration (Potts *et al*. 2000; Pickering *et al*. 2003; Simms *et al*. 2007; Baekey *et al*. 2008). As expected, this was maintained in preparations where the sympathetic drive to the heart had been removed, and was abolished by atropine, confirming the vagal origin of the RSA. RSA was still apparent after unilateral denervation of the heart to permit cardiac vagal branch recordings. This may be attributed to the fact that the cardiac pacemaker receives bilateral vagal inputs in the rat (Sampaio *et al*. 2014). As described previously (Potts *et al*. 2000; Simms *et al*. 2007), direct recordings from the cardiac vagal branch showed a corresponding peak in activity at the onset of post‐inspiration; we found this to occur 0.53 s before the nadir in HR on average. This may be compared with the vagal neuroeffector delay measured at 37°C in adult rats *in vivo* of 200–300 ms (Cividjian *et al*. 2011), although a longer delay is expected in the WHBP because of its lower temperature. Interestingly, although peak cardiac vagal branch firing occurred in post‐inspiration, its activity had already begun to build up during late inspiration. This pattern closely matches that of the vagal inputs to cardiac ganglion cells in the WHBP (McAllen *et al*. 2011), supporting the view that their functional target was indeed the heart. The inspiratory build‐up was also observed in cardiac preganglionic vagal neurons of the nucleus ambiguus *in vivo*, although without the post‐inspiratory surge, which was perhaps suppressed by anaesthesia (Rentero *et al*. 2002). As might be predicted, the post‐inspiratory peak in cardiac vagal activity of the WHBP disappeared when post‐inspiration was abolished by KF inhibition (Dutschmann & Herbert, 2006) and RSA was suppressed. Yet mean cardiac vagal branch activity was not much changed, and substantial chronotropic vagal tone remained.

Neurons in the intermediate part of the KF are essential for the expression of the post‐inspiratory phase (Dutschmann & Herbert, 2006) and their descending respiratory actions are excitatory (Dutschmann & Dick, 2012). These excitatory (glutamatergic) neurons project directly to the region of the nucleus ambiguus (Yokota *et al*. 2015). The simplest interpretation of our findings would thus be that KF post‐inspiratory neurons also send direct excitatory connections to the nucleus ambiguus, driving vagal motoneurons in parallel with post‐inspiration. Tracing studies have identified KF neurons with direct projections to the NA (Song *et al*. 2012), including to subregions that were shown to produce bradycardia on stimulation (Stuesse & Fish, 1984). However, it has not been unequivocally shown that these KF projections innervate cardioinhibitory motoneurons. Indeed, injection of a retrograde transynaptic viral tracer into the cardiac ganglia produced labelling of KF neurons only with comparatively long survival times, suggestive of an intervening synapse (Standish *et al*. 1995). Therefore, we cannot exclude the possibility that interneurons with a post‐inspiratory firing pattern normally mediate the functional link from KF to cardiac vagal motoneurons.

Pontine transection probably removes both inhibitory and excitatory descending inputs to medullary cardiorespiratory nuclei. This may be why pontine transections had quite variable effects on chronotropic vagal tone. The key new finding, however, is that a large component of cardiac vagal tone does not require connections from the pons, and is independent of central respiratory drive. Part of that medullary component depends on neurons in the NTS.

Cardiac vagal tone in mammals has been recognized ever since the 19th century, and is a general feature among vertebrates (Taylor *et al*. 1999). The reflex factors that increase or decrease vagal tone (baroreceptors, chemoreceptors, pulmonary afferents), as well as its modulation by central respiratory drive, have been well studied in mammals, including humans (Anrep *et al*. 1936 *a*; Jewett, 1964; Kunze, 1972; McAllen & Spyer, 1978; Taha *et al*. 1995; Nosaka *et al*. 2000). By contrast, remarkably little is known the central nervous origin of this ongoing activity. Two main reasons may account for this. First, invasive studies on animals *in vivo* typically use general anaesthetics that strongly suppress vagal tone, making it hard to study (Inoue & Arndt, 1982). Second, in isolated *in vitro* preparations such as the transverse brainstem slice, putative vagal preganglionic neurons typically do not generate action potentials (i.e. show no vagal tone) (Mendelowitz, 1996; Dergacheva *et al*. 2010). Decerebrate, unanaesthetized preparations such as the WHBP avoid these drawbacks. The WHBP has a blood‐free environment, which makes interventions such as brainstem transection relatively straightforward. It has robust vagal tone with a physiological pattern. This information provided the starting point for the present study: the sources of cardiac vagal tone appear to be extrinsic to the preganglionic motoneurons but, at least in large part, are intrinsic to the brain stem.

The WHBP is simplified, in that the known reflex sources of cardiac vagal modulation are removed or disabled. Arterial chemoreceptor activity is suppressed by the hyperoxic perfusion medium and the low, non‐pulsatile perfusion pressure provides only minimal drive to arterial baroreceptors, below the cardiac vagal baroreflex threshold (Simms *et al*. 2007). The lack of contribution of these afferents to vagal tone was formally demonstrated here by denervation. There is no need to confirm the absence of lung stretch receptor feedback in the absence of lungs. Additionally, we found that no vagal tone was generated by neurons of the cardiac ganglia. Under these conditions, it is fair to conclude that cardiac vagal tone, and its respiratory modulation, originate in the brainstem.

In the WHBP, the principal pattern of vagal respiratory modulation is a post‐inspiratory peak in activity. In this and a previous study where the pattern of cardiac vagal activity was studied in detail (McAllen *et al*. 2011), we found that it was minimal around the expiratory‐inspiratory transition but was followed by a build‐ up of activity during the latter part of inspiration. In other species, both similar and different patterns have been reported. In the cat, Gilbey *et al*. (1984) found that cardiac vagal motoneurones were subject to a wave of IPSPs that augmented during inspiration. Inspiratory inhibition also appeared to underlie the central component of RSA studied in the dog heart‐lung preparation by Anrep *et al*. (1936 *b*). On the other hand, a progressive rise in vagal excitability during inspiration (measured by the bradycardia following single shocks to the sinus nerve) was observed in anaesthetized dogs by Koepchen *et al*. (1961) and the same pattern was seen in humans in response to brief carotid baroreceptor stimuli applied by neck suction (Eckberg *et al*. 1980). Both excitatory and inhibitory inputs to cardiac vagal motoneurones can evidently contribute to RSA, with similar end results on HR. The excitatory mechanism seen here is present in rats, and may also be present in humans, although it is clearly not the only mechanism of RSA in all species.

### Limitations

The WHBP lacks structures anterior to the midbrain, which can have both excitatory and inhibitory tonic influences on the cardiac vagus (Gellhorn *et al*. 1956; Mauck & Hockman, 1967). It runs at lower than normal body temperature, which serves to amplify vagal actions on HR (Potter *et al*. 1985), although there is no reason to assume that the underlying neural drive is affected very much, or that the lower temperature would somehow recruit *de novo* sources of vagal tone. As proposed elsewhere (Dutschmann *et al*. 2000; St ‐John & Paton, 2003), the eupnoeic, reproducible and stable patterns of respiratory drive in the WHBP, as well as a pronounced pattern of RSA, suggest that brainstem cardiorespiratory generator circuits are behaving normally.

Multifibre activity recorded from the cardiac vagal branch does not simply consist of fibres that control pacemaker function. Those are principally myelinated axons (B fibres) of neurons in the nucleus ambiguus (Cheng *et al*. 2004) and are excited by baroreceptors (Fig. 3). However, recordings almost always include an admixture of fibres probably destined for different regions of the heart or to unknown other targets (O'Leary & Jones, 2003). This factor may explain the imperfect concordance found between the experimental effects on CVBA and HR. Unmyelinated efferent fibres supplying the heart come from neurons in the DMNV (Jones *et al*. 1998); these are generally not barosensitive (Jones *et al*. 1998; O'Leary & Jones, 2003) and synapse with their own distinct subpopulation of cardiac ganglion cells (Cheng *et al*. 2004). Recent studies have shown that the principal function of this pathway is to regulate ventricular function (Machhada *et al*. 2015, 2016): selective inhibition of DMNV neurons in anaesthetized rats caused an increase in ventricular contractility and excitability but no change in HR (Machhada *et al*. 2015, 2016). Presumably, it provides vagal tone to the ventricles but not to the pacemaker, and so any spread to the DMNV of isoguvacine injected into the NTS probably did not confound our conclusions on the vagal control of HR.

To circumvent uncertainties about conclusions from cardiac vagal branch recording, we repeated the experimental series at the same time as measuring chronotropic vagal tone from HR. The results obtained using the two methods were broadly similar, indicating that a substantial component of chronotropic cardiac vagal tone arises independently of RSA. After the influence of pontine structures has been removed, a significant level of chronotropic vagal tone remains and evidently originates within the medulla from sites that include the NTS.

Finally, we can make no firm predictions about the nature of the generators of cardiac vagal tone. For sympathetic nerves, the intrinsic rhythmicity of their signals has been taken as a ‘signature’ of activity generated by central oscillator circuits (Barman & Gebber, 2000). Apart from respiratory modulation, cardiac vagal nerve activity in the WHBP shows no obvious intrinsic rhythmicity. As discussed above, it has been reported that cardiac vagal motoneurons do not appear to possess any intrinsic autoactivity, although the possibility remains that vagal tone is generated by neurons that do. In line with this idea, a select subpopulation of neurons in the medial (‘cardiovascular’) subregion of NTS was found to generate spontaneous pacemaker‐like spike activity *in vitro* (Paton *et al*. 1991). Neurons of this subregion also possess projections to cardiac NA motorneurons (Standish *et al*. 1995; Neff *et al*., 1998). Whether the NTS neurons that reflexly drive cardiac vagal motoneurons in response to baroreceptor and/or chemoreceptor afferent stimulation also show autoactivity is currently unknown, although this would provide a parsimonious neural circuit.

In the clinical setting, cardiac vagal tone is measured non‐invasively by beat‐to‐beat or ‘high frequency’ HR variability. This essentially measures RSA. Although RSA has been found to correlate with vagal tone, it is worth noting that the two measures are not identical and, as reported in the present study, and may have different origins.

## Additional Information

### Competing interests

The authors declare that they have no competing interests.

### Author contributions

DGSF, MD and RMM conceived and designed the experiments. DGSF carried out the experiments, assembled the data and carried out the analysis. All authors contributed to interpretation of the data. DGSF, MD and RMM drafted the article. All authors contributed to critical revision of this article. All authors approved the final version of the manuscript and qualify for authorship. All those who qualify for authorship have been listed. Experiments were carried out at the Florey Institute of Neuroscience and Mental Health.

### Funding

This work was funded by the Australian Research Council (DP130104661) and a grant from the Faculty of Medicine, University of Melbourne. DGSF was funded by the Australian Research Council (DP130104661). JFRP is funded by the British Heart Foundation (RG/12/6/29670). AEP is a Wellcome Trust Senior Clinical fellow (gr088373). MD is supported by an Australasian Research Council Future Fellowship (FT120100953). RMM was funded in part by a Fellowship from the NHMRC (566667). We also acknowledge the support of the Victorian Government through the Operational Infrastructure Scheme.
